# *Lactobacillus murinus* Induces CYP1A1 Expression and Modulates TNF-Alpha-Induced Responses in a Human Intestinal Epithelial Cell Model

**DOI:** 10.3390/ijms262311670

**Published:** 2025-12-02

**Authors:** Husnain Ahmed, Azam A. Sher, Julia A. Bell, Linda S. Mansfield

**Affiliations:** 1 Comparative Enteric Diseases Laboratory, Michigan State University, East Lansing, MI 48824, USA; ahmedhu2@msu.edu (H.A.); sherazam@msu.edu (A.A.S.); bellj@msu.edu (J.A.B.); 2 Comparative Medicine and Integrative Biology Program, Michigan State University, East Lansing, MI 48824, USA; 3 Department of Large Animal Clinical Sciences, Michigan State University, East Lansing, MI 48824, USA; 4 Department of Microbiology and Molecular Genetics, College of Veterinary Medicine, Michigan State University, East Lansing, MI 48824, USA

**Keywords:** *Lactobacillus murinus*, aryl hydrocarbon receptor, CYP1A1 expression, intestinal inflammation

## Abstract

Anti-TNF-α therapy is widely used for inflammatory bowel disease (IBD), but response rates vary, and long-term efficacy declines in many patients. Given the limitations of existing treatments, novel therapeutic strategies are needed. This study investigates whether *Lactobacillus murinus* (*L. murinus*) attenuates tumor necrosis factor alpha (TNF-α)-induced pro-inflammatory responses in a human intestinal epithelial cell model of colitis by modulating the aryl hydrocarbon receptor (AHR). An in vitro model was established using Caco-2 cell monolayers treated with TNF-α to simulate intestinal inflammation. Cells were pre-treated with *L. murinus* or known AHR ligands, and the effects on AHR activation, barrier integrity, and inflammatory response were assessed via transepithelial electrical resistance (TEER) and IL-8 quantifications. As CYP1A1 is a well-established transcriptional target of AHR, its mRNA expression was used as a surrogate marker of AHR modulation in this model. TNF-α stimulation significantly disrupted epithelial barrier integrity and increased IL-8 secretion in a dose-dependent manner. *L. murinus* pre-treatment enhanced CYP1A1 expression and was associated with reduced TNF-α-induced barrier disruption and IL-8 secretion. Notably, the beneficial effects of *L. murinus* on epithelial integrity were not replicated by synthetic AHR ligands, suggesting ligand-selective differences in AHR related responses. These findings suggest that AHR-associated signaling induced by *L. murinus* may contribute to mitigation of TNF-α-induced epithelial barrier dysfunction and inflammation. This study identifies a potential probiotic-associated mechanism that warrants further investigation, including studies designed to establish a causal role of AHR dependency in the observed effects. In addition, future studies are needed to identify the specific *L. murinus* metabolites responsible for inducing CYP1A1 expression and activating the AHR pathway.

## 1. Introduction

Inflammatory bowel disease (IBD) encompasses chronic inflammatory conditions affecting the gastrointestinal (GI) tract, primarily categorized as Crohn’s disease (CD) and ulcerative colitis (UC) [1,2]. The prevalence of IBD continues to rise, with an estimated 1 in 209 adults in the United States diagnosed, and approximately 70,000 new cases reported annually [3]. CD can affect any segment of the GI tract, leading to transmural inflammation, strictures, and fistula formation, whereas UC is characterized by continuous mucosal inflammation confined to the colon [4].

Conventional treatment strategies for IBD include aminosalicylates, immunosuppressants, and monoclonal antibodies [5]. Among biologics, infliximab, the first FDA-approved anti-TNF-α monoclonal antibody, has significantly altered the therapeutic landscape of IBD [6,7]. However, up to 30% of patients exhibit primary non-response, while nearly 50% of initial responders experience loss of efficacy (secondary non-response) [8,9]. Additionally, long-term anti-TNF-α therapy poses risks, including increased susceptibility to infections and malignancies, prompting the need for safer and more effective treatment alternatives [9].

IBD pathogenesis is multifactorial, involving genetic susceptibility, environmental factors, immune dysregulation, and gut microbiota alterations [10]. Genetic studies have identified mutations in the *NOD2* gene that increase susceptibility to CD [11]. Furthermore, alterations in gut microbiota composition such as a reduced abundance of Firmicutes and an increased abundance of pro-inflammatory bacteria have been implicated in IBD development [12,13]. Given the link between gut dysbiosis and IBD, probiotics have emerged as potential therapeutic agents to restore microbial balance and modulate inflammation [14]. Although some probiotic formulations have shown clinical efficacy in mild-to-moderate UC, their precise mechanisms of action remain unclear [15]. Identifying specific bacterial strains that exert protective effects and elucidating their underlying molecular pathways are critical for advancing microbiome-based therapies.

One emerging pathway of interest is the aryl hydrocarbon receptor (AHR), a ligand-activated transcription factor involved in epithelial barrier maintenance and immune regulation [16,17]. Studies suggest that certain bacterial metabolites can modulate the AHR pathway, leading to anti-inflammatory effects, yet the direct interaction between putative probiotics such as *L. murinus* and AHR remains underexplored [18,19].

In this study, we investigated whether *L. murinus*, a putative probiotic strain, can influence AHR signaling and mitigate TNF-α-induced epithelial dysfunction in an in vitro colitis model. To address this, Caco-2 monolayers grown on Transwell inserts were used to simulate the human intestinal epithelium. Monolayers were pre-treated with either *L. murinus* or AHR ligands before being challenged with TNF-α, a pro-inflammatory cytokine known to disrupt epithelial barrier function [20,21]. Barrier integrity was assessed through transepithelial electrical resistance (TEER) measurements, while IL-8 was quantified as a marker of intestinal inflammatory responses [22]. Additionally, AHR modulation was evaluated by measuring CYP1A1 mRNA expression, a widely accepted biomarker of AHR activity [23,24,25]. We hypothesized that *L. murinus* would increase CYP1A1 expression, thereby activating the AHR-associated pathway, and that this response would attenuate TNF-α-induced epithelial inflammation and barrier disruption in vitro.

## 2. Results

### 2.1. Caco-2 Monolayers Form Robust Tight Junctions When Cultured on Transwell Inserts

**Figure 1:**
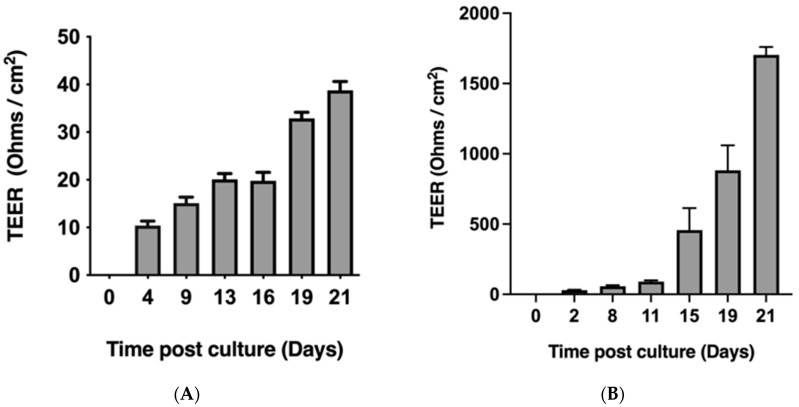
Resistance readings of the HT-29 and Caco-2 monolayers. TEER of the HT-29 (**A**) and Caco-2 (**B**) monolayers was measured when grown on the Transwell^TM^ inserts. The monolayers were grown on the Transwell^TM^ insert for the period of 21 days. The resistance was measured every couple of days. Resistance readings are shown as mean ± SEM from three independent biological replicates (n = 3). Note: Statistical analysis was not performed for the TEER measurements, as the primary purpose of this figure is to illustrate the trend in epithelial barrier development over time. The data are presented to demonstrate that Caco-2 cells achieved a TEER value exceeding the physiological threshold of 500 Ω·cm^2^, indicative of functional monolayer formation, whereas HT-29 cells did not reach this threshold under the same culture conditions.

Caco-2 [26] and HT-29-MTX-E12 [27] cell lines were initially selected to model the human intestinal epithelial barrier in vitro. Monolayers of each cell line were cultured separately on Transwell inserts for 21 days. TEER was measured between the apical and basolateral compartments of the Transwell system to assess tight junction formation. A TEER value of 500 ohms·cm^2^ was used as the threshold indicative of functional tight junctions [28]. By day 21, Caco-2 monolayers exhibited TEER values significantly exceeding 500 ohms·cm^2^ (Figure 1B), indicating the formation of robust tight junctions. In contrast, HT-29-MTX-E12 cells demonstrated much lower TEER values, averaging approximately 40 ohms·cm^2^ by day 21 (Figure 1A), suggesting poor tight junction integrity. Based on these results, Caco-2 cells were selected for further experiments as a reliable in vitro model of the human intestinal epithelial barrier due to their ability to form strong and consistent tight junctions when grown on Transwell inserts.

### 2.2. TNF-α Reduces Intestinal Epithelial Barrier Integrity in a Concentration-Dependent Manner

**Figure 2:**
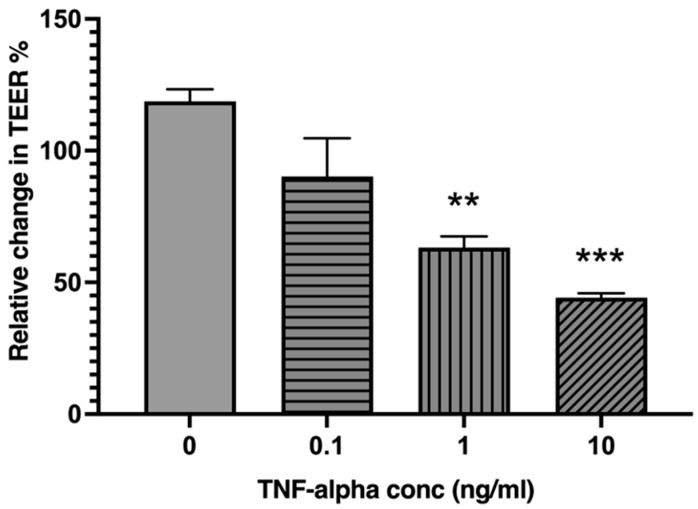
TNF-α decreases epithelial barrier integrity in a concentration-dependent manner. Caco-2 cell monolayers were treated with 10-fold increasing concentrations of TNF-α. TEER was measured at 24 h post TNF-α stimulation. Relative TEER% was calculated as the percent change of TEER at 0 h. The results are expressed as mean ± SEM. There were at least 3 technical replicates per group. The statistical analysis was performed using one-way ANOVA (F(3,8) = 16.72, *p* = 0.0008) with post hoc Tukey’s HSD test for computing statistically significant differences relative to the control group (0 ng/mL). ** *p* < 0.01; *** *p* < 0.001.

Exposure to increasing concentrations of TNF-α caused a concentration-dependent reduction in TEER (Figure 2). Treatment with 0.1 ng/mL TNF-α produced a small decrease in TEER that was not statistically significant compared with the untreated control (*p* = 0.1273). In contrast, when compared to untreated control (0 ng/mL), 1 ng/mL TNF-α caused a significant reduction in TEER (*p* = 0.0050), and 10 ng/mL TNF-α induced the greatest decrease (*p* = 0.0007). Overall, these findings demonstrate that TNF-α impairs epithelial barrier integrity in a concentration-dependent manner.

### 2.3. TNF-α Does Not Alter the mRNA Expression of Occludin and Tight Junction Protein 1 (TJP-1) in a Concentration-Dependent Manner

**Figure 3:**
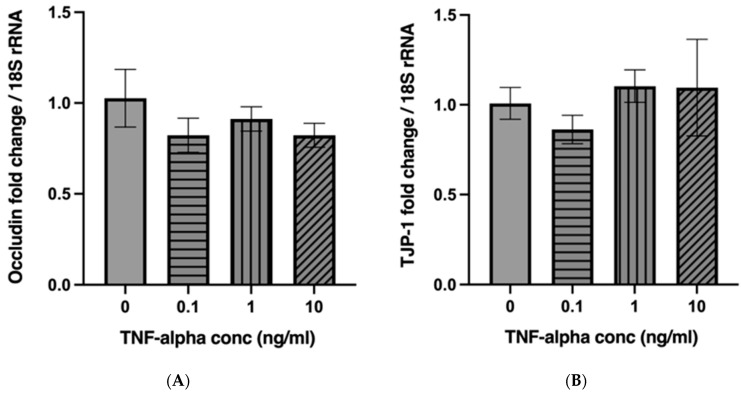
TNF-α does not affect the mRNA expression of Occludin and TJP-1. Caco-2 cell monolayers were treated with 10-fold increasing concentrations of TNF-α. The mRNA of occludin (**A**) and TJP-1 (**B**) was quantified by qRT-PCR using 18S rRNA as an internal control. The results are expressed as mean ± SEM. There were at least 3 technical replicates per group. Statistical analysis was conducted using one-way ANOVA, which showed no significant differences among groups for either occludin (F(3,8) = 0.8720, *p* = 0.4947) or TJP-1 (F(3,8) = 0.5283, *p* = 0.6752).

The mRNA expression levels of occludin and tight junction protein 1 (TJP-1) were quantified by qRT-PCR, using 18S rRNA as the internal control (Figure 3A,B). Increasing concentrations of TNF-α did not significantly affect the mRNA expression of either occludin (*p* = 0.4947) or TJP-1 (*p* = 0.6752). The data suggest that TNF-α exposure does not alter the transcription of occludin and TJP-1.

### 2.4. TNF-α Induces IL-8 Secretion from Intestinal Epithelial Cells in a Concentration-Dependent Manner

**Figure 4:**
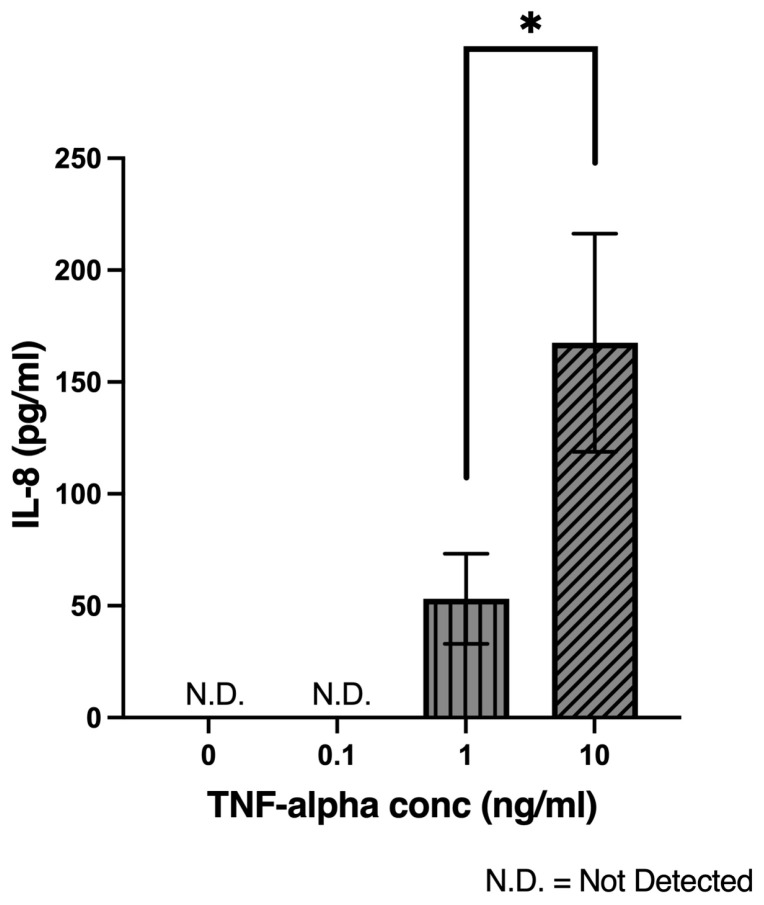
TNF-α induces IL-8 secretion from Caco-2 monolayers in a concentration-dependent manner. The Caco-2 monolayers were treated with increasing concentrations of TNF-α for 24 h. IL-8 protein was quantified by ELISA from the cell culture medium collected from the basal chamber of the Transwell inserts. There were at least 3 technical replicates per group. N.D indicates “not detected”, as IL-8 protein levels were below the detection threshold of the ELISA kit. Statistical analysis comparing 1 ng/mL and 10 ng/mL TNF alpha was performed using a one-tailed unpaired Student’s *t*-test (t(4) = 2.170, *p* = 0.0479). * *p* < 0.05.

The concentration-dependent effect of TNF-α on IL-8 secretion was evaluated in Caco-2 monolayers. Cells were treated with increasing concentrations of TNF-α (0.1, 1, and 10 ng/mL) for 24 h. No detectable IL-8 protein was observed in the untreated control group (0 ng/mL TNF-α) or in cells treated with 0.1 ng/mL TNF-α. In contrast, a robust IL-8 secretion was observed in cells treated with 1 ng/mL and further increased at 10 ng/mL of TNF-α. A direct comparison between the 1 ng/mL and 10 ng/mL groups confirmed a statistically significant increase in IL-8 production at the higher concentration (*p* = 0.0479). These findings indicate that TNF-α induces IL-8 secretion in a concentration-dependent manner once concentrations exceed 1 ng/mL (Figure 4).

### 2.5. TCDD and FICZ Increase CYP1A1 Transcription in a Concentration-Dependent Manner

After establishing an in vitro colitis model by stimulating Caco-2 cells with TNF-α, we investigated whether AHR could be modulated by its well-defined exogenous and endogenous ligands in this system. TCDD was selected as a prototypical exogenous synthetic AHR agonist [29], while FICZ, a tryptophan-derived photoproduct generated under ultraviolet radiation, was chosen as it represents the most potent known endogenous AHR ligand [30].

Caco-2 cells were treated for 12 h with increasing concentrations of TCDD (0.1 nM, 1 nM, and 10 nM) or FICZ (1 nM, 10 nM, and 100 nM). Following treatment, AHR modulation was assessed by quantifying CYP1A1 mRNA expression, a well-established transcriptional target and biomarker of AHR activation [31].

**Figure 5:**
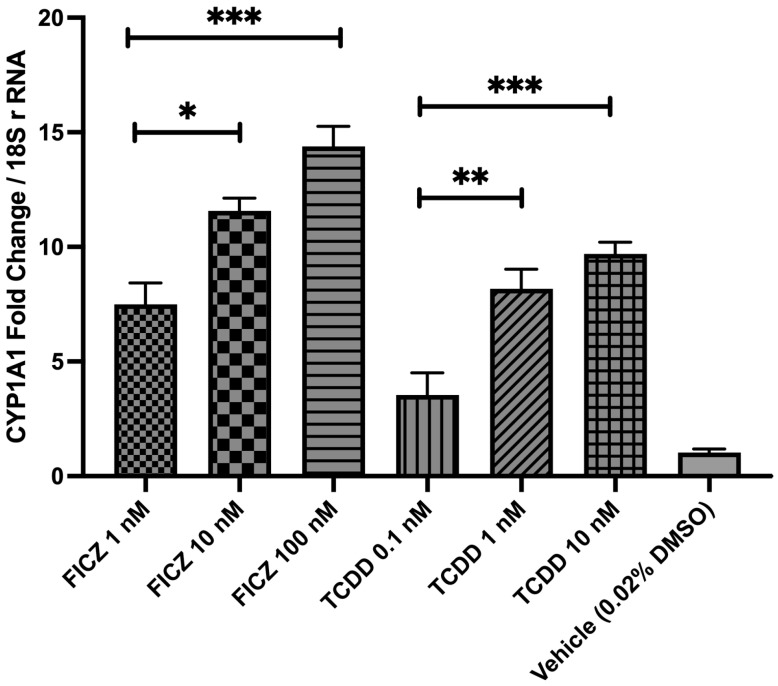
AHR ligands increase CYP1A1 transcription in a concentration-dependent manner. The expression of CYP1A1 was measured in the Caco-2 cells treated with different concentrations of the two defined AHR agonists, i.e., FICZ and TCDD, for 12 h. The expression of the CYP1A1 was determined by qRT-PCR and the results were normalized using 18S rRNA as an internal control. The results are expressed as mean ± SEM. There were at least 3 technical replicates per group. The statistical analysis between different groups was performed using one-way ANOVA (F(6,14) = 37.63, *p* < 0.0001) with post hoc Tukey’s HSD test for comparing multiple treatments. * *p* < 0.05; ** *p* < 0.01; *** *p* < 0.001.

Treatment with FICZ produced a clear concentration-dependent increase in CYP1A1 transcription (Figure 5). A modest induction was observed at 1 nM, followed by significantly higher expression at 10 nM (*p* = 0.0218) and 100 nM (*p* = 0.0002) relative to 1 nM FICZ. TCDD also increased CYP1A1 transcription in a concentration-dependent manner, with higher concentrations producing markedly greater expression. Relative to 0.1 nM TCDD CYP1A1 transcription increased significantly at 1 nM (*p* = 0.0084) and was further elevated at 10 nM (*p* = 0.0007). Vehicle control (0.02% DMSO) produced minimal CYP1A1 expression, consistent with baseline values.

### 2.6. Lactobacillus murinus Modulates the CYP1A1 Transcription in Multiplicity of Infection (MOI)-Dependent Manner

**Figure 6:**
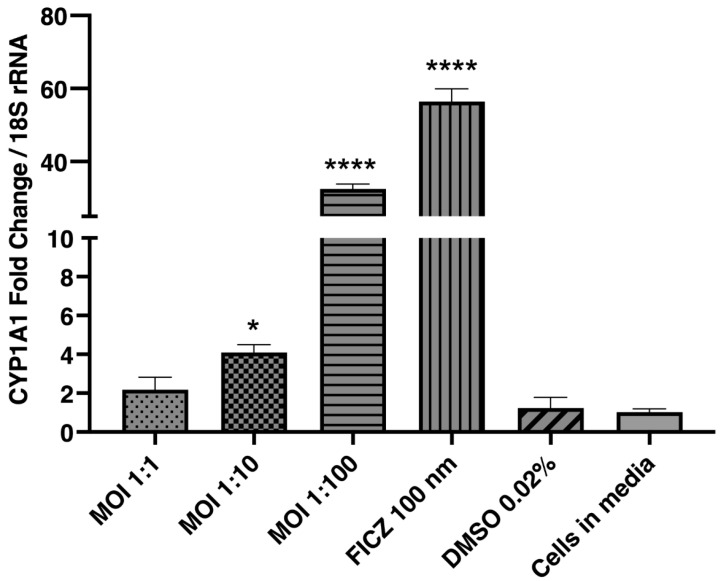
*L. murinus* upregulates CYP1A1 transcription in an MOI-dependent manner. Caco-2 cells were treated with *L. murinus* in 10-fold-increasing MOI. CYP1A1 activity was measured by qRT-PCR with 18S rRNA used as an internal control. Cells were treated with 100 nm FICZ as positive control and 0.02% DMSO as vehicle control. All treatments were given for 24 h. There were at least 3 technical replicates per group. The results are expressed as mean ± SEM. The statistical analysis between different groups was performed using one-way ANOVA (F(5,12) = 660.5, *p* < 0.0001) with post hoc Tukey’s HSD test for comparing multiple treatments. * *p* < 0.05; **** *p* < 0.0001.

After determining that AHR can be modulated in the Caco-2 cells by its defined agonists, we then sought to determine if *L. murinus* can modulate the AHR-pathway in Caco-2 cells. To determine this, Caco-2 monolayers were exposed to *L. murinus* across 10-fold-increasing MOIs for 24 h, followed by qRT-PCR analysis. CYP1A1 mRNA levels increased in an MOI-dependent pattern, with a modest elevation at MOI 1:10 (*p* < 0.05) and a markedly higher response at MOI 1:100 (*p* < 0.0001) compared to the vehicle control. The results suggest that *L. murinus* increased CYP1A1 transcription in an MOI-dependent manner (Figure 6).

### 2.7. AHR Associated Signaling Attenuated TNF-α-Induced Gut Barrier Disruption and Pro-Inflammatory Response in Caco-2 Monolayers

Next, we asked if AHR modulation can attenuate TNF-α-induced decrease in TEER and IL-8 secretion in Caco-2 monolayers. To determine this, we first modulated the AHR pathway in the Caco-2 monolayers by pre-treating with TCDD (10 nm), FICZ (100 nm) or *L. murinus* (MOI 1:100). After 12 h of pre-treatment, the monolayers were then stimulated with TNF-α (10 ng/mL) for a period of 24 h. Relative TEER percentage and IL-8 protein were quantified as a measure of efficacy of the pre-treatment.

**Figure 7:**
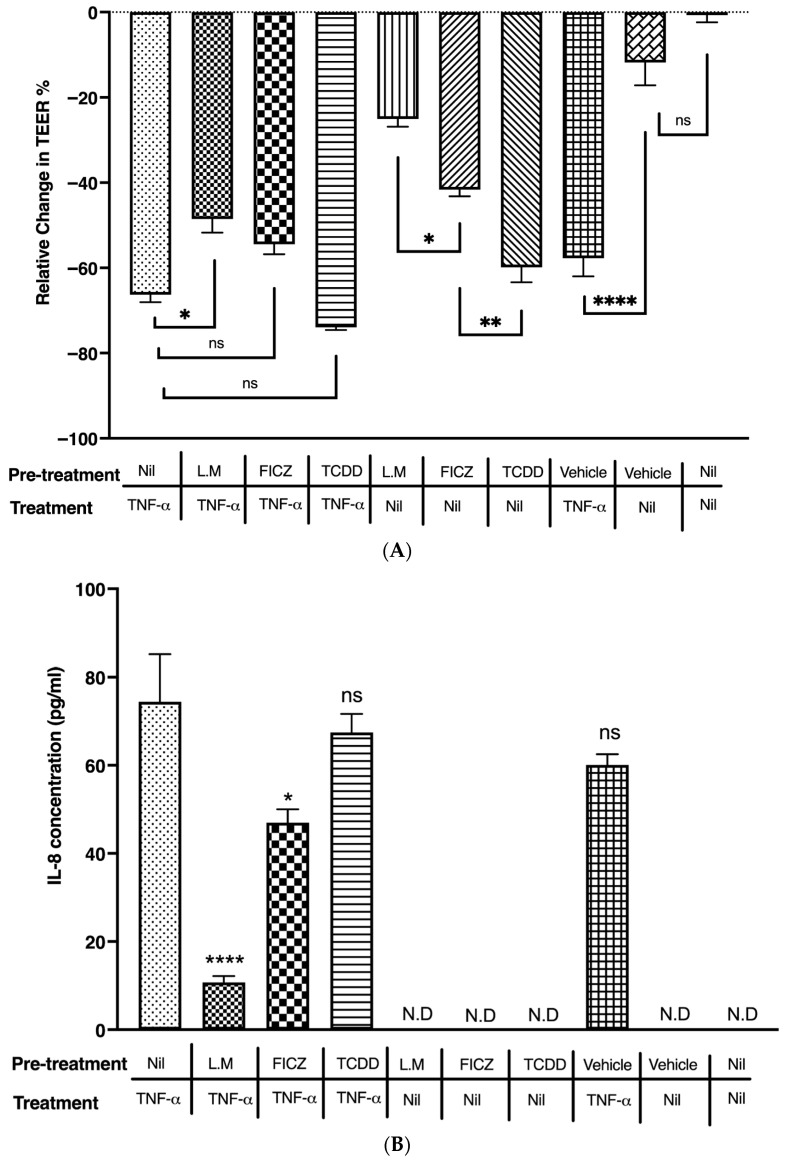
AHR activation attenuates epithelial barrier disruption and pro-inflammatory response in Caco-2 monolayers. Caco-2 monolayers were given respective pre-treatments for 12 h with either *L. murinus* (MOI 1:100) or FICZ (100 nM) or TCDD (10 nM), followed by 24 h of treatment with TNF-α (10 ng/mL). At the end of 24 h of treatments, TEER and IL-8 were measured to determine the effect of AHR activation on barrier dysfunction (**A**) and IL-8 secretion (**B**) from Caco-2 cells. The results are expressed as mean ± SEM. There were at least 3 technical replicates per group (n = 1). In (**B**), “N.D” indicates “not detected” as IL-8 levels were below the detection limit of the ELISA kit. Statistical analysis for relative change in TEER (**A**) was performed using one-way ANOVA (F(9,20) = 66.0, *p* < 0.0001) followed by Tukey’s post hoc test for multiple comparisons. The statistical analysis for IL-8 concentration (**B**) was performed using one-way ANOVA (F(4,10) = 21.0, *p* < 0.0001) followed by Dunnett’s multiple comparisons test, comparing each pre-treatment group to the TNF-alpha control. * *p* < 0.05; ** *p* < 0.01; **** *p* < 0.0001.

*L. murinus* pre-treatment was associated with the attenuation of TNF-α-induced TEER disruption. However, pre-treating the monolayers with TCDD or FICZ was not effective in attenuating the TNF-α-induced epithelial barrier damage (Figure 7A). These results suggest that *L. murinus* pre-treatment was effective in attenuating the TNF-α-induced damage to the intestinal epithelial barrier function in vitro.

There was a significant increase in IL-8 production upon the treatment of monolayers with TNF-α (10 ng/mL). However, *L. murinus* pre-treatment significantly reduced the secretion of IL-8 upon TNF-α stimulation (*p* < 0.0001). Furthermore, modulating the AHR pathway by FICZ also significantly reduced IL-8 production upon TNF-α stimulation (*p* = 0.0176). However, pre-treating the monolayers with TCDD did not reduce the IL-8 secretion when stimulated with TNF-α (Figure 7B). These results suggest that modulation of the AHR-pathway by some but not all AHR ligands attenuates the TNF-α-induced pro-inflammatory response in the human intestinal epithelial cells.

## 3. Discussion

IBD is characterized by chronic inflammation and disruption of the intestinal epithelial barrier, where pro-inflammatory cytokines such as TNF-α play a central role in disease pathogenesis. Current therapies, including anti-TNF-α biologics, are effective but have significant limitations, including loss of response over time and increased risk of infections. These challenges have prompted interest in alternative strategies aimed at restoring epithelial barrier integrity and attenuating excessive inflammation [2,3].

In this study, we used a human intestinal epithelial cell model (Caco-2 monolayers) to investigate whether *L. murinus* can modulate inflammatory responses induced by TNF-α in association with modulation of the AHR pathway. Our results suggests that *L. murinus* increases CYP1A1 expression, consistent with modulation of AHR pathway, in an MOI-dependent manner and attenuates TNF-α-induced IL-8 secretion and barrier disruption, as evidenced by preservation of TEER. These findings suggest a potential mechanism by which specific probiotic strains might exert protective effects on the intestinal epithelium, potentially through modulation of AHR pathways.

AHR is a ligand-activated transcription factor known for its role in xenobiotic metabolism but increasingly recognized for its regulatory effects on intestinal barrier homeostasis and immune responses [32,33]. In our study, both exogenous (TCDD) and endogenous (FICZ) AHR ligands induced CYP1A1 expression, confirming functional AHR activation in Caco-2 cells. Notably, *L. murinus* elicited similar CYP1A1 induction, supporting its potential role as a microbial modulator of AHR-associated signaling.

Pre-treatment with *L. murinus* partially preserved epithelial barrier function following TNF-α exposure. However, pre-treatment with TCDD or FICZ did not demonstrate a similar protective effect on TEER. One possible explanation for these differential outcomes lies in the ligand-selective activation profiles of these AHR ligands. TCDD is a highly stable compound that resists AHR-induced metabolism, resulting in prolonged and sustained activation of AHR signaling [34]. In contrast, FICZ, an endogenous tryptophan-derived ligand, is rapidly metabolized by cytochrome P450 enzymes, leading to transient activation of AHR [30,35]. These differences in activation kinetics may influence the balance between beneficial and detrimental downstream signaling. Sustained AHR activation by TCDD has been shown to dysregulate homeostatic pathways and induce toxicity in various cell types [36]. Although we did not assess downstream pathways in this study, previous work has shown that different AHR ligands can differentially influence signaling networks including activation of mitogen-activated protein kinase (MAPK) pathways, which have been linked to barrier disruption in epithelial cells [20,37]. The paradoxical finding in our study, where TCDD and higher concentrations of FICZ did not protect or even worsened TEER, may therefore reflect ligand-specific transcriptional responses rather than a uniform effect of AHR activation. While TEER provides a sensitive and widely used functional readout of tight junction-dependent ionic permeability, it does not capture macromolecular flux or structural junctional integrity. We complemented TEER with mRNA analysis of the tight junction genes occludin and TJP1; however, additional assays such as FITC dextran permeability or immunofluorescence imaging of tight junction proteins will be important in future studies to more comprehensively characterize barrier function. These observations highlight the complexity of AHR-mediated responses in epithelial cells and underscore the importance of considering ligand-specific dynamics when evaluating AHR as a potential therapeutic target.

Interestingly, while FICZ pre-treatment reduced IL-8 secretion in response to TNF-α stimulation, TCDD did not have a similar effect. This finding further emphasizes the ligand-selective nature of AHR-mediated transcriptional regulation. As previously discussed, different AHR ligands have been shown to elicit distinct gene expression profiles, depending on their chemical properties, receptor binding affinity, metabolic stability, and the cellular context in which they act [38,39]. Endogenous ligands like FICZ typically lead to transient and tightly regulated AHR activation, favoring anti-inflammatory and homeostatic gene expression programs, such as the upregulation of barrier-protective factors and suppression of pro-inflammatory cytokines [40,41]. In contrast, synthetic and persistent ligands like TCDD may promote sustained activation that results in dysregulation of immune responses or cytotoxicity. The divergent effects on IL-8 secretion observed in our study may reflect such differences in AHR ligand behavior. While FICZ appears to engage AHR signaling pathways that suppress IL-8 production, TCDD may either fail to trigger these same anti-inflammatory pathways or actively induce opposing signals that sustain or exacerbate IL-8 expression. Furthermore, the long half-life and poor metabolism of TCDD lead to its prolonged presence in cells, which may reflect ligand-selective differences in AHR transcriptional output [34].

In this study, IL-8 was selected as the primary inflammatory marker due to its robust and reproducible induction by TNF-α in intestinal epithelial cells and its role as a potential biomarker for UC [22]. However, we acknowledge that measuring IL-8 alone does not capture the full breadth of epithelial inflammatory signaling. Additional cytokines and chemokines, such as IL-6, MCP-1, or GM-CSF, were not assessed in the present study. Future work incorporating a broader inflammatory panel will be important for fully characterizing how *L. murinus* and distinct AHR ligands influence epithelial immune responses.

Although this study suggests that *L. murinus* increases CYP1A1 expression, consistent with AHR modulation, we did not employ AHR specific antagonists (e.g., CH-223191) [42]. This is because the inhibitory activity of CH-223191 is ligand-dependent and preferentially inhibits the halogenated aromatic hydrocarbon class of agonists (e.g., TCDD) but not others, such as polyaromatic hydrocarbons or flavonoids [43]. As this study did not identify the specific AHR agonists produced by *L. murinus*, using CH-223191 in this study could have yielded inconclusive or misleading results. However, definitive testing of AHR dependency will require AHR knockdown, knockout, or CRISPR-based approaches in future studies.

Additionally, while our results suggest AHR modulation by *L. murinus*, the specific metabolites or molecular components responsible for this activation were not identified. Future studies are needed to identify the specific bioactive metabolites responsible for this AHR-associated activation. Liquid chromatography–mass spectrometry (LC–MS)-based metabolomic profiling of *L. murinus*-conditioned media will be an essential next step for future studies to identify the AHR agonists produced by *L. murinus*.

In summary, our findings suggest that *L. murinus* can increase CYP1A1 expression in Caco-2 cells and may contribute to mitigating TNF-α–induced pro-inflammatory responses and barrier disruption. These results provide initial mechanistic insight into how *L. murinus* may influence AHR-associated signaling, as reflected by increased CYP1A1 transcription. They also offer a foundation for future work, including studies that establish a causal role of AHR dependency for the observed effects and identify the specific *L. murinus* metabolites responsible for increased CYP1A1 transcription and modulation of AHR signaling.

## 4. Materials and Methods

### 4.1. Chemicals and Reagents

2,3,7,8-Tetrachlorodibenzo-p-dioxin (TCDD), dissolved in dimethyl sulfoxide (DMSO), was purchased from AccuStandard Inc. (New Haven, CT, USA). 6-Formylindolo[3,2-b] carbazole (FICZ) was obtained from Sigma-Aldrich (St. Louis, MO, USA; Catalog No. SML1489). DMSO (Catalog No. D8418) was also sourced from Sigma-Aldrich. Recombinant human tumor necrosis factor-alpha (TNF-α) protein (Catalog No. 210-TA-005) was purchased from R&D Systems (Minneapolis, MN, USA).

### 4.2. Bacterial Culture

The *Lactobacillus murinus* strain was originally isolated from C57BL/6 IL-10^−^/^−^ mice. The methods used for its isolation and characterization have been previously described by Brudvig et al. [44,45]. Briefly, *L. murinus* was cultured on De Man, Rogosa, and Sharpe (MRS) agar plates (Neogen^®^, Lansing, MI, USA) by streak plating. The plates were incubated at 37 °C under microaerophilic conditions (5% CO_2_) for 48 h. After incubation, bacterial colonies were harvested and suspended in sterile phosphate-buffered saline (PBS) (Millipore-Sigma, Burlington, MA, USA; Catalog No. 806552). The optical density at 600 nm (OD_600_) was measured using a spectrophotometer. The relationship between OD_600_ values and colony-forming units per milliliter (CFU/mL) was previously established by plating serial dilutions on MRS agar and enumerating the resulting colonies. The bacterial suspension was then adjusted to an OD_600_ of approximately 1.0, corresponding to 5 × 10^6^ CFU/mL, and subsequently added to the Caco-2 cell culture medium.

### 4.3. Cell Culture

Caco-2 cells were obtained from the American Type Culture Collection (ATCC, Gaithersburg, MD, USA) and used between passages 6 and 10. Cells were cultured in Minimum Essential Medium Eagle (Millipore-Sigma, Burlington, MA, USA; Catalog No. M2279) supplemented with 20% fetal bovine serum (FBS; Rocky Mountain Biologics, Missoula, MT, USA; Catalog No. FBS-CBT), 1% L-glutamine (Thermo Fisher Scientific, Waltham, MA, USA; Catalog No. 25030-081), and 1% MEM non-essential amino acid solution (Thermo Fisher Scientific; Catalog No. 11140050). Undifferentiated Caco-2 cells were cultured on 24-well tissue culture plates (Alkali Scientific, Fort Lauderdale, FL, USA; Catalog No. TPN 1024) at a seeding density of 5 × 10^4^ cells per well. Differentiated Caco-2 cells were cultured on Transwell inserts (Corning Life Sciences, Durham, NC, USA) at a seeding density of 5 × 10^4^ cells per insert to model the intestinal epithelial barrier.

HT-29-MTX-E12 cells were originally purchased from Sigma-Aldrich (St. Louis, MO, USA) and were used between passages 51 and 60. These cells were maintained in Dulbecco’s Modified Eagle Medium (DMEM; Gibco™, Jenks, OK, USA; Catalog No. 11995073), supplemented with high glucose (4500 mg/L), sodium pyruvate (110 mg/L), 10% FBS (Rocky Mountain Biologics, Missoula, MT, USA; Catalog No. FBS-CBT), 1% L-glutamine (Thermo Fisher Scientific; Catalog No. 25030-081), and 1% MEM non-essential amino acids solution (Thermo Fisher Scientific; Catalog No. 11140050).

### 4.4. Cell Treatments

All treatments were administered for 12 h, except for TNF-α, which was used to stimulate the cells for 24 h. Caco-2 monolayers were treated with increasing concentrations of TCDD (0.1 nM, 1 nM, and 10 nM) and FICZ (1 nM, 10 nM, and 100 nM), which served as positive controls for AHR activation. Vehicle control cells were treated with DMSO at a final concentration of 0.02% in the culture medium.

### 4.5. Measurement of TEER

Caco-2 cells were seeded on 6.5 mm Transwell inserts from Corning Life Sciences, Durham, NC, USA (Catalog No. 3470) at a density of 5 × 10^4^ cells per insert. Transepithelial electrical resistance (TEER) was measured using an EVOM2 epithelial volt/ohm meter from World Precision Instruments, Sarasota, FL, USA to evaluate monolayer integrity. Monolayers were considered to have formed functional tight junctions once TEER values reached 500 ohms per square centimeter or higher. TEER measurement is a widely accepted method for assessing the integrity and confluence of tight junctions in Caco-2 cell monolayers [46,47].

### 4.6. Quantification of IL-8 Protein by Enzyme-Linked Immunosorbent Assay (ELISA)

Following 24-h treatment with TNF-α, cell culture medium was collected from the basal chamber of the Transwell inserts. IL-8 protein levels were quantified using an IL-8 ELISA kit (Invitrogen, Waltham, MA, USA; Catalog No. BMS 204-3) according to the manufacturer’s protocol. Optical density (OD) was measured at 450 nm using a microplate reader (Bio-Tek, Winooski, VT, USA). Sample concentrations were determined based on a standard curve generated from the known controls provided with the kit.

### 4.7. Quantitative Reverse Transcription PCR

Total RNA was extracted using the RNeasy Mini Kit (Qiagen, Germantown, MD, USA; Catalog No. 74104) following the manufacturer’s instructions. RNA purity was assessed by measuring absorbance ratios with a NanoDrop ND-1000 spectrophotometer (Wilmington, DE, USA). Samples with an A260/280 ratio of approximately 2.0 and an A260/230 ratio near 2.2 were considered sufficiently pure for downstream analysis. A total of 300 ng of RNA was used for complementary DNA (cDNA) synthesis with the High-Capacity cDNA Reverse Transcription Kit (Applied Biosystems, Waltham, MA, USA; Catalog No. 4368814).

Quantitative real-time PCR (qRT-PCR) was performed using TaqMan Universal PCR Master Mix (Applied Biosystems; Catalog No. 4304437) along with TaqMan gene expression assays for human CYP1A1 (Hs00153120-m1; Catalog No. 4453320), 18S rRNA (Hs03003631-g1; Catalog No. 4448489), tight junction protein 1 (TJP1; Hs01551871-m1; Catalog No. 4448892), and occludin (Hs00170162-m1; Catalog No. 4453320). Reactions were run on a Quant Studio 3 Real-Time PCR System (Applied Biosystems).

Thermal cycling conditions included an initial denaturation step at 95 °C for 10 min, followed by 40 cycles of denaturation at 95 °C for 15 s and annealing/extension at 60 °C for 1 min. Relative gene expression was calculated using the ΔΔCt method [48].

### 4.8. Statistical Analysis

Graphical representations and statistical analyses were generated using GraphPad Prism version 9.2.0, with data presented as mean ± standard error of the mean (SEM). The Kolmogorov–Smirnov test was conducted to assess the normality of data distribution. Comparisons between two groups were performed using an independent samples Student’s *t*-test. For comparisons involving more than two groups, one-way analysis of variance (ANOVA) was used. Bartlett’s test was applied to evaluate the homogeneity of variances prior to conducting ANOVA. Statistical significance was indicated as follows: ns (not significant) *p* > 0.05; * *p* < 0.05; ** *p* < 0.01; *** *p* < 0.001; **** *p* < 0.0001.

## 5. Conclusions

This study suggests that *L. murinus* increases CYP1A1 expression in intestinal epithelial cells, consistent with modulation of AHR signaling, and is associated with reduced TNF-α-induced IL-8 secretion and barrier disruption in vitro. These findings suggest that *L. murinus* may influence epithelial responses to inflammatory stimuli through modulation of an AHR-related pathway. Overall, this work provides an initial framework for exploring AHR-associated mechanisms through which probiotics may contribute to epithelial barrier protection and modulate epithelial inflammatory responses.

## Data Availability

The original contributions presented in this study are included in the article. Further inquiries can be directed to the corresponding author.
